# Real-Time Continuous Surveillance of Temperature and Flow Events Presents a Novel Monitoring Approach for Hospital and Healthcare Water Distribution Systems

**DOI:** 10.3390/ijerph16081332

**Published:** 2019-04-13

**Authors:** Harriet Whiley, Jason Hinds, James Xi, Richard Bentham

**Affiliations:** 1 College of Science and Engineering, Flinders University, GPO Box 2100, Adelaide, SA 5001, Australia; Richard.Bentham@flinders.edu.au; 2 Enware Australia Pty Ltd., 11 Endeavour Road, Caringbah, NSW 2229, Australia; Jason.Hinds@enware.com.au (J.H.); James.Xi@enware.com.au (J.X.)

**Keywords:** *Legionella*, *Pseudomonas*, non-tuberculous mycobacteria, healthcare, opportunistic waterborne pathogens, water quality, water management, public health

## Abstract

Within hospitals and healthcare facilities opportunistic premise plumbing pathogens (OPPPs) are a major and preventable cause of healthcare-acquired infections. This study presents a novel approach for monitoring building water quality using real-time surveillance of parameters measured at thermostatic mixing valves (TMVs) across a hospital water distribution system. Temperature was measured continuously in real-time at the outlet of 220 TMVs located across a hospital over a three-year period and analysis of this temperature data was used to identify flow events. This real-time temperature and flow information was then compared with microbial water quality. Water samples were collected randomly from faucets over the three-year period. These were tested for total heterotrophic bacteria, *Legionella* spp. and *L. pneumophila*. A statistically significant association with total heterotrophic bacteria concentrations and the number of flow events seven days prior (*rs*[865] = −0.188, *p* < 0.01) and three days prior to sampling (*rs*[865] = −0.151, *p* < 0.01) was observed, with decreased heterotrophic bacteria linked to increased flushing events. Only four samples were positive for *Legionella* and statistical associations could not be determined; however, the environmental conditions for these four samples were associated with higher heterotrophic counts. This study validated a simple and effective remote monitoring approach to identifying changes in water quality and flagging high risk situations in real-time. This provides a complementary surveillance strategy that overcomes the time delay associated with microbial culture results. Future research is needed to explore the use of this monitoring approach as an indicator for different opportunistic pathogens.

## 1. Introduction

Opportunistic premise plumbing pathogens (OPPPs) are an increasingly significant public health issue [1,2,3]. In hospitals, it is argued that water distribution systems are the most important but overlooked source of hospital-acquired infections [3]. Faucets and water distribution systems have been recognised as reservoirs and sources of infection for a range of pathogens including *Legionella* spp., *Pseudomonas aeruginosa*, *Aeromonas* spp., *Acinetobacter* spp., *Burkholderia* spp., *Enterobacter* spp., *Flavobacterium* spp., *Serratia marcescens*, *Stenotrophomonas maltophilia* and non-tuberculous mycobacteria (NTM) [3,4,5]. Increasing aged populations and their associated elevated risk susceptibility are driving the need for innovative approaches to monitoring and managing the risk from OPPPs in order to better protect public health [6].

An established source of OPPPs in hospital and healthcare water distribution systems is the municipal potable water supplies [7,8]. Once microorganisms enter a system, they form, or are incorporated into, biofilm—a matrix of extracellular organic polymers combined with inorganic particles [4]. Biofilm build-up and the corrosion of pipeline surfaces is a primary cause of decreased water quality [9]. Biofilm formation also provides a nutrient source and protection from unfavourable environmental conditions [10]. Subsequent detachment of biofilms provides a mechanism for further distribution, colonization, or transmission of pathogens, potentially resulting in human infection [11].

Numerous variables impact biofilm formation, including water temperature, disinfection type and residual, pipe materials and sizes, as well as temporal changes in water hydraulics and chemistries [2]. One of the most significant indicators of biofilm formation is fluctuating water usage and temporal stagnation [12,13,14]. Incorrectly balanced water hydraulics have been demonstrated to promote significant bacterial growth [10]. A previous study of household drinking water found that overnight stagnation resulted in a 2–3-fold increase of cell concentrations [13]. Another study of an office building found that during the first year of operation, stagnation resulted in significant deterioration of water quality and increase in viable biomass [15]. The combined effects of loss of temperature control and loosening of attached or suspended biofilms can result in rapid removal and dissemination when flow is re-established [12]. The prevention of stagnation in hot water systems has been recognised to reduce the risk of *Legionella pneumophila* in potable water distribution systems [9].

In Australia, the delivery of hot water is regulated according to the National Building Code that captures the Australian Standard AS/NZS 3500. The standard sets required temperatures for storage and delivery of heated water to outlets. The standard also specifies the temperature of water at outlets on the basis of the risk to occupants and their vulnerability. This standard encompasses both minimisation of microbial growth and the potential for scalding of users [16].

Installation of thermostatic mixing devices to deliver water at temperatures that minimise risk of scalding is an intrinsic part of the regulation. The necessity to deliver water at temperatures favourable to the survival and colonisation of OPPPs is an unavoidable risk of regulatory compliance. A global strategy in controlling this risk is to frequently flush outlets to remove biofilm debris and introduce residual disinfection [17].

Current regulations require the use of microbial water testing to inform the management and treatment of warm water systems. However, there are many limitations associated with the standard culture method of detection, including the underestimation of viable but non-culturable (VBNC) bacteria and the time delay from sampling to results. As such, there is a need for real-time monitoring strategies which will better inform risk management protocols [6]. This study investigated the novel approach of monitoring real-time temperature fluctuations in thermostatic mixing valves (TMVs) located in an Australian hospital that were linked to flow events and their relation to microbial water quality. The association with TMV average daily temperature, the number and duration of flushing events on the day and up to seven days prior to sampling with concentrations of total heterotrophic bacteria and *Legionella* spp. were explored.

## 2. Materials and Methods

### 2.1. Study Site

The study site was a hospital building less than 5 years old located in Sydney, NSW, containing 125 inpatient rooms spread over 12 floors. During the study period from 12 April 2013 until 22 May 2017, the temperature of water output from 220 TMVs was constantly monitored and 800 water samples were collected over the study period. The data presented in this study was collected as part of the hospital’s regulatory requirements and provides real world insight into the information used to manage water quality within hospital water distribution systems.

### 2.2. Temperature

The temperature of water outgoing from 220 TMVs located across the building was continuously monitored using a thermocouple temperature sensor hardwired back to an electronic water management system. The sensor was located just below the TMV outlet in a specially designed mixing chamber that ensured blending of the mixed water to improve temperature recording accuracy. This enabled every 0.5 °C temperature change to be recorded continuously. This was then reported as average daily temperature 7 days prior, 3 days prior and then on the day of sampling and microbial analysis.

### 2.3. Flushing Events

As described above, water temperature data was continuously collected. An algorithm (see Supplementary Figure S1) was then used to identify and separate flow events based on changes in temperature. These were broken into flow/flushing events greater or less than 15 s duration. The collected data was separated into different categories according to the flushing frequency.

Total flushing events on day of sampling,
Total flushing events less than 15 s duration on day of sampling,
Total flushing events longer than 15 s on day of sampling,
Total flushing events during the 3 days prior to sampling,
Total flushing events during the 7 days prior to sampling.

### 2.4. Microbiological Sampling

A total of 865 water samples were collected from faucets attached to TMVs as part of the hospital’s mandatory verification monitoring program in accordance with NSW Code of Practice for the Control of Legionnaires’ Disease (2004). Microbial analysis was conducted by a National Association of Testing Authorities Australia (NATA) accredited laboratory. Total heterotrophic bacteria were enumerated on plate count agar using AS/NZS 4276.3.1:2007; *Legionella* spp. (other than *L. pneumophila*), *L. pneumophila* SG1 and *L. pneumophila* serogroup 2–14 were enumerated on BCYE-GVPC agar in accordance with AS 3896:2008.

### 2.5. Statistics

Statistical analysis was conducted using SPSS version 25.0 (IBM, NY, USA). A Kolmogorov–Smirnov test, Q-Q plot and histogram demonstrated that the data was not normally distributed. A linear mixed model was used to determine if there was a need to control for nesting of data due to tap location needs. The intraclass correlation coefficient was 2.4%, therefore limited variability was due to TMV location and the microbial data was treated as independent. Scatterplots were created and the loess fit line was used to visualise the association between variables. This was followed by calculating the non-parametric correlation coefficient. A bootstrap solution for simple linear regression was used to examine the three-way association between the variables.

The distribution of measurements was examined using a chi-squared goodness of fit. This showed a bias in the distribution of measurement due to the higher frequency of sampling during November and December. However, due to the increase in the cold water temperature during these months (Australian summer), they pose the greatest risk for microbial growth.

## 3. Results

A total of 629/865 (73%) of samples collected were positive for total heterotrophic bacteria at a range of 1–52,000. The temperature of the water at the sampling locations ranged from 25.0 °C to 89.0 °C, however, the median temperature was 25.7 °C. The number of flow/flushing events on the day of sampling ranged from 0–730 for events less than 15 s in duration, 0–639 for events greater than 15 s in duration and 0–1369 total events.

**Figure 1:**
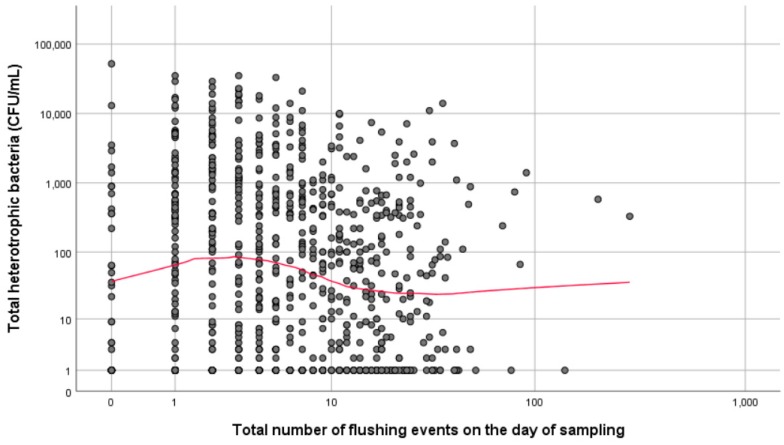
Simple scatter plot with loess line of fit showing the association between total heterotrophic bacteria (colony forming units (CFU)/mL) and total number of flushing events on the day of sampling.

As the number of flushing/flow events on the day of sampling increased there was an initial increase in total heterotrophic bacteria observed. This could possibly be attributed to biofilm detachment caused by sheering [18]. However, after five flushing events there was a decline in total heterotrophic bacteria, which plateaued after around 10 flushing events (Figure 1). The Spearman’s rho revealed that the relationship between the total number of flushing event (*rs*[865] = −0.106, *p* < 0.01) and total heterotrophic bacteria was statistically significant. This was also true for total number of flushing events greater than 15 s (*rs*[865] = −0.126, *p* < 0.01), but not the total number of flushing events less than 15 s (*rs*[865] = −0.017, *p* = 0.609).

**Figure 2:**
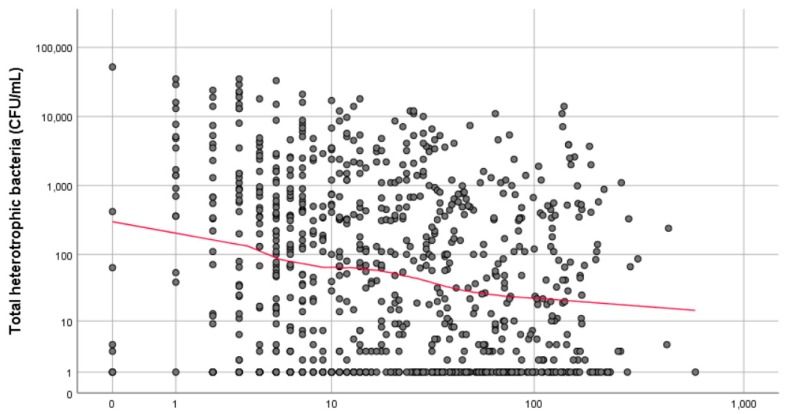
Simple scatter plot with loess line of fit showing the association between total heterotrophic bacteria (CFU/mL) and the total number of flushing events during the 7 days prior to sampling.

The Spearman’s rho revealed a statistically significant relationship between the concentration of total heterotrophic bacteria and the number of flushing events seven days prior to sampling *(rs*[865] = −0.188, *p* < 0.01) and three days prior to sampling (*rs*[865] = −0.151, *p* < 0.01), with increased flow events associated with decreased concentrations of heterotrophic bacteria (Figure 2). The most significant relationship was observed with the number of flushing events during the 7 days prior to sampling.

**Figure 3:**
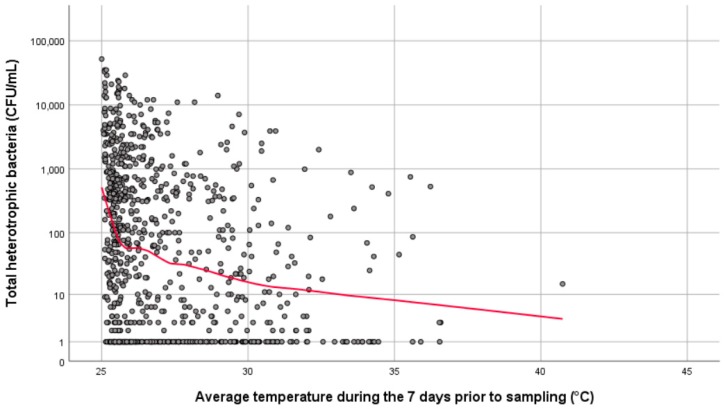
Simple scatter plot with loess line of fit showing the association between total heterotrophic bacteria (CFU/mL) and average water temperature (°C) during the 7 days prior to sampling.

There was also a relationship between temperature and the total heterotrophic bacteria, with lower temperatures associated with higher heterotrophic bacteria (Figure 3). Spearman’s rho revealed a statistically significant association between total heterotrophic bacteria and average temperate on the day of sampling (*rs*[865] = −0.238, *p* < 0.01) and average temperature for the 7 days prior to sampling (*rs*[865] = −0.258, *p* < 0.01).

During the sampling period, only 4/865 (0.5%) samples were positive (limit of detection 10 colony forming units (CFU)/mL) for *L. pneumophila* and all four were serogroup 1. No samples were positive for *Legionella* spp. other than *L. pneumophila*. All four water samples were also positive for total heterotrophic bacteria, and concentrations ranged from 690 CFU/mL to 14,000 CFU/mL. The number of flow events and average temperatures for the sampling events which were positive for *L. pneumophila* are shown in Table 1. The average temperature during the 7 days prior to sampling ranged from 25 °C to 30 °C. This temperature range was also associated with higher numbers of total heterotrophs. Two of the sampling sites had 11 and 17 flushing events during the 7 days prior to sampling; however, sample site 45 had 136 and 139 flushing events during the 7 days prior to sampling.

## 4. Discussion

This study presents a novel approach for monitoring building water quality using real-time surveillance of temperature and flow events. This surveillance approach was validated by the statistically significant association of increased heterotrophic bacteria observed at sampling points with few flushing events and low temperatures.

The association of increased heterotrophic bacteria with stagnant water is supported by previous research [13,14]. An investigation into biofilm formation in a model distribution system found that increased stagnation promoted biofilm formation. This relationship was also observed in a study of household faucets which found that overnight stagnation induced microbial growth [13]. Another study showed that the taps in homes that were very infrequently used had the highest bacterial contamination. Stagnation increased microbial numbers in water from those taps which were otherwise frequently used [14]. Although in this study there were not enough *Legionella*-positive samples for statistical analysis, previous studies have demonstrated *Legionella* concentrations to be positively associated with total heterotrophic bacteria at 22 °C and 37 °C, but not at temperatures above 55 °C [19]. Additionally, a study conducted in 1984 demonstrated that stagnant hot water storage tanks in a hospital were positive for *Legionella* and were seeding the distribution system.

This study also found that there was an association between increased flushing events and increased water temperatures. In this hospital distribution system, the warm water ranged from 25.0 °C to 89.0 °C, and increased bacteria concentrations were associated with lower water temperatures. This is supported by a study of Quebec hospitals conducted in 1992 which demonstrated that increased *Legionella* concentrations were statistically significantly (*p* = 0.03) associated with low water temperatures at faucet [20]. Additionally, a recent study using a model reticulating water system confirmed that increased temperatures were critical for controlling *L. pneumophila* growth [21].

There is an observed relationship between the influences of flushing events and temperatures at faucets. The ability to remotely monitor both of these factors that contribute to microbial colonisation and dissemination is of more relevance. This study validated that a remote monitoring strategy could be used to ensure good thermal control and minimisation of stagnation at outlets within potable water systems. The biggest advantage with the real-time monitoring strategy in this study is that it can be used to complement, and reduce the reliance on, microbial testing. The detection of pathogens and other microbiological indicators is typically conducted off site, which is costly, time consuming and increases the risk of cross-contamination or loss of culturability [22,23].

## 5. Conclusions

This study demonstrated the use of temperature sensors located at the outlet of TMVs as a novel surveillance approach for hospital and healthcare water distribution systems. The temperature and flow event information provided an effective indicator of total heterotrophic bacteria in the water. This could provide an efficient means of identifying high-risk areas within the water distribution system in real time, and overcome the limitations associated with microbial testing.

## Figures and Tables

**Table 1:** Temperature, flow events and total heterotrophic bacteria counts for samples which were positive for *L. pneumophila* serogroup 1.

Sample Location ID	Date	Average Temperature (°C)		Number of Flushing Events		Microbiological Results (CFU/mL)	
		On the Day of Sampling	During the 7 Days Prior to Sampling	On the Day of Sampling	During 7 Days Prior to Sampling	Total Heterotrophic Bacteria	*Legionella pneumophila* SG1
58	15 November 2013	26.9	25.4	10	11	690	10
45	31 January 2014	29.9	28.2	31	136	11,000	10
45	26 June 2014	31.1	29	36	139	14,000	20
63	25 September 2014	28	26.1	7	17	3500	20

## Supplementary Materials

The following are available online at https://www.mdpi.com/1660-4601/16/8/1332/s1. Figure S1: Flow/flushing event algorithm.
