# Supplementary material for: Real-Time Continuous Surveillance of Temperature and Flow Events Presents a Novel Monitoring Approach for Hospital and Healthcare Water Distribution Systems
Source: Int J Environ Res Public Health. 2019 Apr 13;16(8):1332. doi: 10.3390/ijerph16081332 (PMC6518245; doi:10.3390/ijerph16081332)
Supplement: Supplementary file 1 [file ijerph-16-01332-s001.pdf]

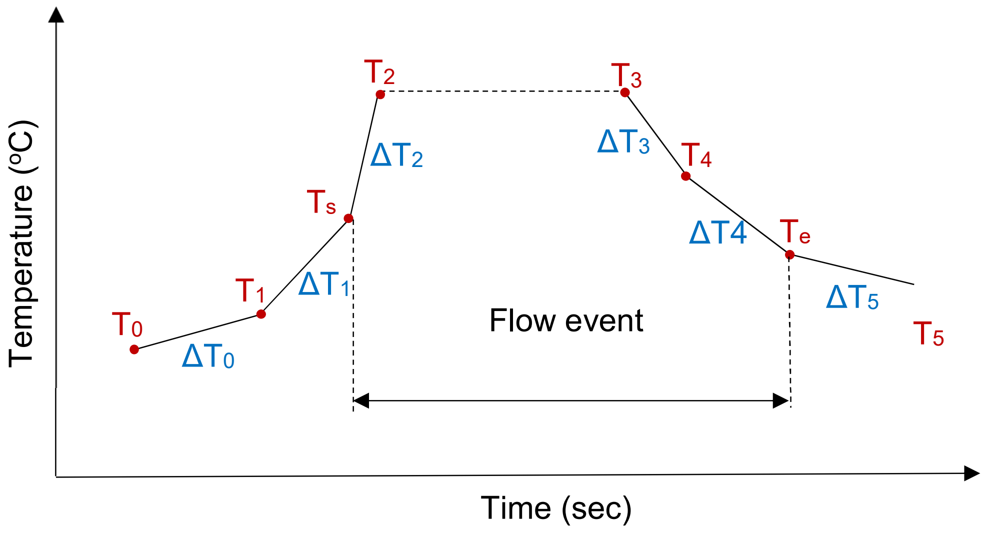

<u>Legend</u>

When searching for the start of a flow event

T_s = current temperature

T_2 = temperature after T_s

T_1 = temperature prior to T_s

T_0 = temperature prior to T_1

When searching for the end of a flow event

T_e = current temperature

T_5 = temperature after T_e

T_4 = temperature prior to T_e

T_3 = temperature prior to T_4

<u>Algorithm:</u>

Status = no flow event

For each temperature in records do:

- If status = no flow event then:
  - read T_s, T_0, T_1, T_2
  - If $\Delta T_0 \geq 0$ and $\Delta T_1 \geq 0$ and $\Delta T_2 \geq 0$ and $T_2 - T_0 > \max(\Delta T_0, \Delta T_1, \Delta T_2)$ then:
    - Status = flow event
    - Continue and go to next temperature
- If status = flow event then:
  - read T_e, T_3, T_4, T_5
  - If $\Delta T_3 \leq 0$ and $\Delta T_4 \leq 0$ and $\Delta T_5 \leq 0$ and $T_5 - T_3 < \min(\Delta T_3, \Delta T_4, \Delta T_5)$ then:
    - Status = no flow event

Figure S1. Flow/flushing event algorithm.
